# Analysis of a Tubular Torsionally Resonating Viscosity–Density Sensor

**DOI:** 10.3390/s20113036

**Published:** 2020-05-27

**Authors:** Daniel Brunner, Joe Goodbread, Klaus Häusler, Sunil Kumar, Gernot Boiger, Hassan A. Khawaja

**Affiliations:** 1 Institute of Computational Physics, Zurich University of Applied Sciences, ZHAW, 8400 Winterthur, Switzerland; boig@zhaw.ch; 2 Faculty of Engineering Science and Technology, Department of Automation and Process Engineering, The Arctic University of Norway, UiT, P.O. Box 6050 Langnes, 9037 Tromsø, Norway; hassan.a.khawaja@uit.no; 3 Rheonics GmbH, 8406 Winterthur, Switzerland; klaus.haeusler@rheonics.com (K.H.); sunil.kumar@rheonics.com (S.K.)

**Keywords:** viscometer, viscosity–density sensor, viscosity measurement, torsional resonator, fluid–structure interaction

## Abstract

This paper discusses a state-of-the-art inline tubular sensor that can measure the viscosity–density (ρη) of a passing fluid. In this study, experiments and numerical modelling were performed to develop a deeper understanding of the tubular sensor. Experimental results were compared with an analytical model of the torsional resonator. Good agreement was found at low viscosities, although the numerical model deviated slightly at higher viscosities. The sensor was used to measure viscosities in the range of 0.3–1000 mPa·s at a density of 1000 kg/m^3^. Above 50 mPa·s, numerical models predicted viscosity within ±5% of actual measurement. However, for lower viscosities, there was a higher deviation between model and experimental results up to a maximum of ±21% deviation at 0.3 mPa·s. The sensor was tested in a flow loop to determine the impact of both laminar and turbulent flow conditions. No significant deviations from the static case were found in either of the flow regimes. The numerical model developed for the tubular torsional sensor was shown to predict the sensor behavior over a wide range, enabling model-based design scaling.

## 1. Introduction

Traditionally, viscosity is measured by sampling and analyzing fluids with common laboratory viscometers or rheometers. These instruments are time consuming, error prone, expensive, and prohibit a fast and automated system response. Sensors based on mechanical resonance, however, are a promising alternative to conventional laboratory equipment. These sensors are robust, have no moving parts, and are, therefore, suited to real-time measurements. Using sensors based on resonance, various materials can be investigated for different purposes, such as studying the viscoelastic behavior of polymers, determining fluid density and viscosity [1,2,3,4,5,6,7,8], characterizing the mechanical properties of polymer membranes and thin films [9,10,11,12,13,14], and detecting biomolecule or nanoparticle masses [15,16,17,18,19,20]. Sensors using torsional vibration are a subgroup of mechanical resonators. If purely cylindrical, these sensors create pure shear stresses and do not increase mass displacement, such as tuning forks or cantilevers. This makes them more robust, and measurement less sensitive towards, e.g., wall effects. 

Sensors based on torsional vibration have been investigated to measure viscous and viscoelastic effects [21,22,23,24]. Probe-style sensors are already commercially available (e.g., Rheonics, Hydramotion, Sofraser, Brookfield, and Emerson). Experimental and numerical studies have been conducted on how to measure viscosity [25,26,27,28,29]. Furthermore, they have been used to characterize viscoelastic fluids [21]. 

It is advantageous to have a nonintrusive viscosity sensor to monitor industrial processes. Thereby, the sensor should neither create an obstruction in the piping system nor influence the flow field inside the tube. 

Häusler and Reinhart et al. [26,30] designed a tubular sensor on the basis of a small tube to measure blood viscosity. The sensor consisted of a small tube with an inner diameter of 2 mm that was excited in a torsional mode. The damping of the mode was measured and correlated to fluid viscosity within the tube. This system was used to measure blood viscosity at different hematocrits.

Fuchs and Drahm et al. [31] built a tubular sensor to measure the mass flow rate, fluid density and viscosity. The sensor was based on a straight tube with an attached eccentric mass. The system oscillated in a superposition of torsional and transverse modes, which allowed the Coriolis effect to be measured. In addition, due to fluid displacement, the resonance frequency of the excited mode could be correlated with fluid density. The novelty in this design was that the sensor could measure the damping of the mode. Damping is caused by the shear stresses within the fluid due to torsional vibration. Thus, the sensor was capable of measuring the mass flow rate, viscosity, and density.

This study presents a tubular sensor that could measure the viscosity (at a known density) under the conditions of internal flow. The design is based on the tubular sensor introduced by Häusler [26,30]. It was adapted to measure a wide range of viscosities. Additionally, the sensor was designed as a flow-through device, which can be integrated into piping systems and does not obstruct the flow. The resonator of the sensor oscillates in a purely torsional mode; thus, it cannot measure flow rate or fluid density like the tubular sensor designed by Fuchs and Drahm [31]. However, because no eccentric mass is needed, the overall inertia of the resonator is smaller than that with eccentric mass. Thus, the ratio between fluid-induced damping and inertia is higher, and the sensor is more sensitive towards damping. This enables greater accuracy, especially for low viscosities. Therefore, the benefit of the new tubular sensor is higher accuracy at low viscosities in comparison to the tubular sensor presented by Fuchs and Drahm [31].

To gain deeper insight into the working principle of the sensor, the sensor was numerically modelled using a weak fluid–structure interaction. This model will provide the means for dimensional scaling of the sensor while meeting sensor’s measuring range and accuracy specifications. For validation, the predicted damping values were verified by comparing them with measurements under static conditions, meaning no internal flow and thermally uniform conditions. However, the sensor eventually operated under conditions where internal flow is present. Thus, it was crucial to investigate the sensitivity of measurement to internal flow to reliably and accurately conduct measurements to reflect actual industrial use case. Therefore, the sensor was inserted into a flow loop, and tested with different fluids and in the laminar and turbulent flow regime. 

## 2. Sensor Design and Experiments

The tubular sensor uses a thin-walled, straight, stainless-steel tube as the sensor body. The fluid flows through the tubular sensor without any interruption. This allows the tubular sensor to be directly integrated into a process line.

The working principle of the tubular sensor is based on torsional resonance. The first torsional mode of the tubular resonator is excited at a frequency similar to its natural frequency. The excited resonance creates motion in the fluid. The shear stresses caused by the fluid motion induce a torque on the sensor, which damps oscillation. Oscillation damping is measured and related to fluid properties. 

### 2.1. Tubular Sensor Design

**Figure 1:**
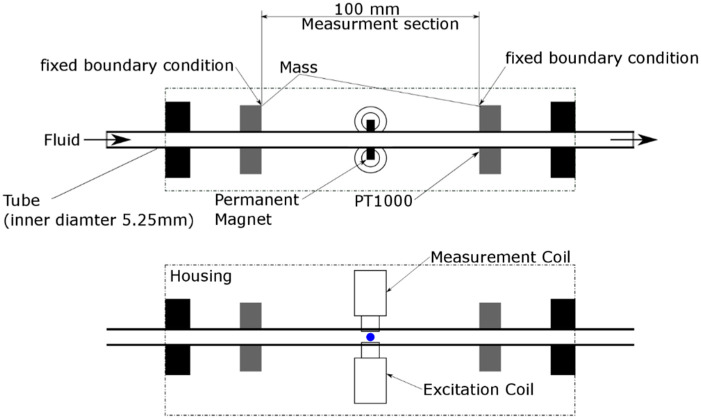
Experimental setup of tubular sensor.

The schematic of the tubular sensor is shown in Figure 1. The resonator was comprised of a thin-walled, stainless-steel (316 L) tube with an inner diameter of 5.25 mm and with two large disks mounted onto the outer diameter. The two disks are spaced 100 mm apart. The section between these disks is the measurement section, where the first torsional mode was excited via two permanent magnets that are mounted onto the tube. These magnets were driven by electromagnets, which produced an oscillating torque near the natural frequency of the first torsional mode. This driving torque was turned off after sufficient energy has been provided to the resonator. Then, the decay in torsional oscillation was measured using electromagnets. On the basis of the measured signal, the logarithmic decrement and the resonance frequency $f_{0}$ of the resonator were computed. Damping was expressed as bandwidth  Γ, which was computed on the basis of the logarithmic decrement. Additionally, temperature was measured by a PT1000 RTD (Honeywell, Berkshire, UK) mounted on the tube.

### 2.2. Static Experiment Procedure

Experiments were conducted under static conditions (tube filled with fluid with no internal flow) to determine damping at different well-defined viscosities and densities of the fluid. These experiments were used to determine whether the numerical model described in Section 2.4 agrees with the measurement as well as to check consistency for different fluids. To conduct the experimental measurements, the tube was filled with different NIST (National Institute of Standards and Technology) traceable viscosity reference fluids (N2, S6, S20, S60, N100, S200, and S600, from Cannon Instrument Company (State College, PA 16803, USA)). These fluids have a well-known viscosity and density as a function of temperature; thus, they are well suited for calibration and validation purposes. During calibration, the temperature varied between 20 and 100 °C. Once a target temperature was reached, it was held constant for long enough so that the sensor and fluid were under thermally uniform conditions.

The damping caused by the fluid is related to the product of viscosity and density, later denoted as ρη value, where ρ is the fluid density and η the dynamic viscosity. Each fluid covers a certain range of ρη values. However, all fluids are of similar density, and therefore, the driving change in damping is related to the fluid’s viscosity. These ranges overlap; thus, two fluids are capable of producing the same damping (ρη value) at different temperatures. 

**Figure 2:**
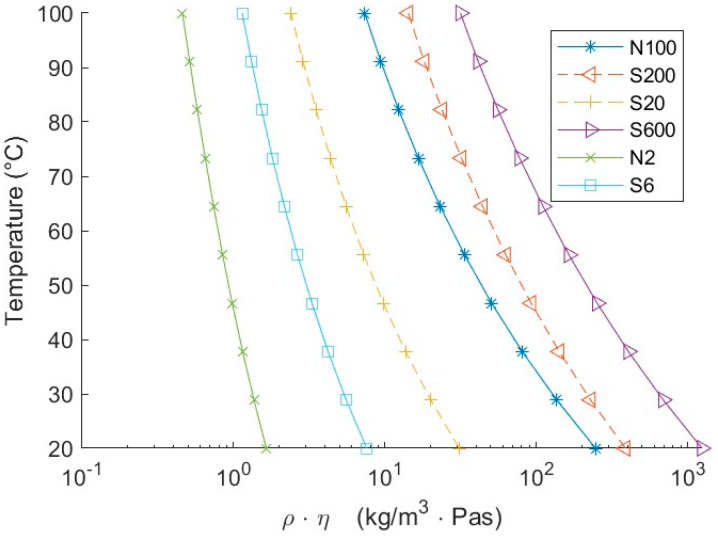
Product of viscosity and density (ρη) of fluids as a function of temperature. Solid lines are fluids used to create a baseline, and dashed lines are fluids used for validation.

This investigation was conducted in two different steps. In the first step, four fluids were used to create a baseline for the sensor. These first sets of fluids are marked in Figure 2 with full lines. They covered the entire operating range of the sensor and had some overlap of their temperature-dependent ρη value. In the second step, baseline validity was tested with additional fluids S20 and S200, marked with dashed lines in Figure 2. 

During measurement, sensor damping and resonance frequency were determined. The measured damping of the sensor was the superposition of intrinsic material damping and fluid-induced damping. To determine fluid-induced damping, intrinsic damping was subtracted from the measured damping value. The intrinsic damping of the sensor was temperature dependent and measured prior to fluid measurements. Therefore, the clean sensor with no fluid inside the tube was measured with the same protocol as the filled sensor in the climate chamber.

For all measurements, sensor bandwidth was measured in intervals of approximately 1 s. One hundred measurements were used to calculate an averaged value of bandwidth, temperature, and resonance frequency. To estimate measurement uncertainty, error estimation was performed. There were two main contributions to the error: (1) intrinsic damping and (2) measuring damping value.

(1) An absolute error in the measured damping was caused by the intrinsic damping of the sensor. This error was independent of the damping value.
(2) Measurement of the damping value was more accurate at low damping due to higher signal-to-noise ratio. The relative error was 0.3% in air and increased to 30% for viscosities of 1000 mPas at a density of 1000 kg/m³. This error could be reduced by averaging multiple measurements. Thus, by averaging 100 measurements, its contribution was reduced by ten-fold.

To determine the absolute viscosity (at a given density), the exact fluid properties at a given temperature during measurements were required. Temperature measurement was subject to its own error, creating uncertainty around the fluid properties during measurements. For the fluids used in this study, this error was approximately 3%.

### 2.3. Flow Loop Experiment

**Figure 3:**
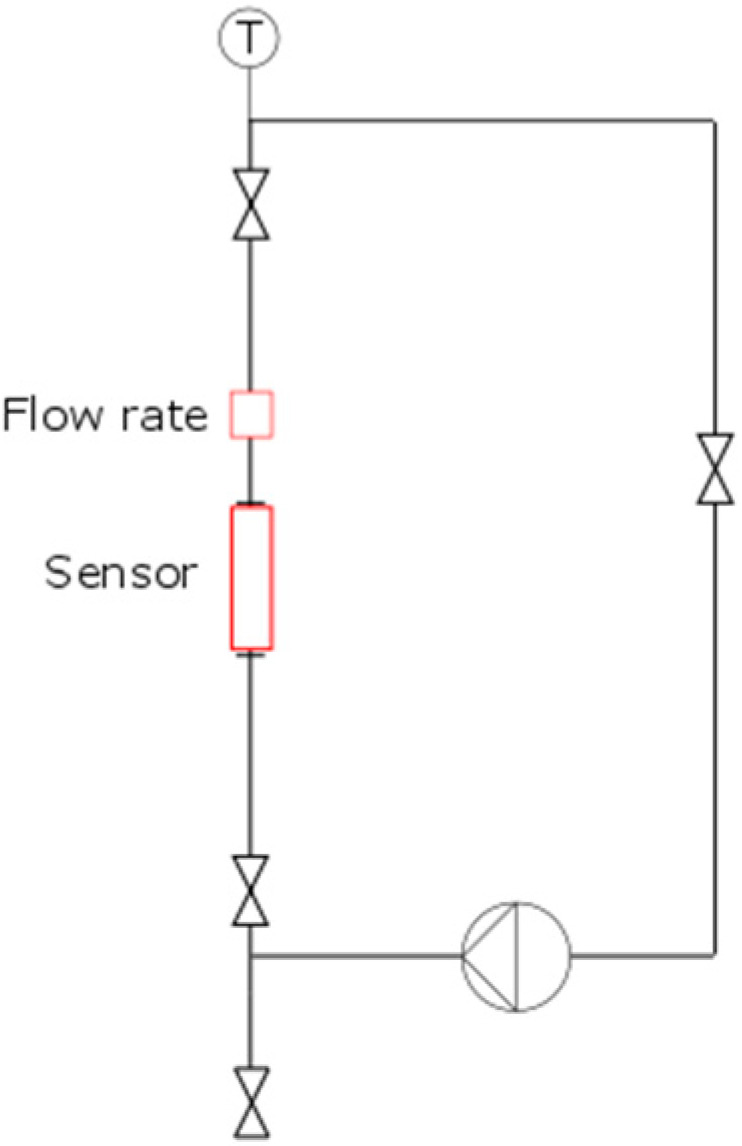
Flow loop schematic used for internal flow experiments (Reynolds number range of 500–50,000).

The tubular sensor was integrated into a flow loop to investigate the sensor sensitivity towards internal flow under realistic industrial conditions (as shown in Figure 3). Flow rate could be varied in the flow loop, allowing variation in the averaged flow velocity through the sensor from 2.3 to 10 m/s. A membrane pump (ZIP-80, Wagner (Altstätten, Switzerland)) was used to circulate the fluid, creating a pulsating flow. The flow rate was measured after the tubular sensor. Experiments were conducted at room temperature (27–32 °C) with a water–glycerol solution at 10 different concentrations (83%–8.3% W-G). Viscosities varied between 1 and 45 mPas at a density of approx. 1000 kg/m^3^. At each concentration, five measurements at different flow rates were taken. These five measurements were compared to the static measurements (flow rate = 0).

### 2.4. Resonator Modeling

**Figure 4:**
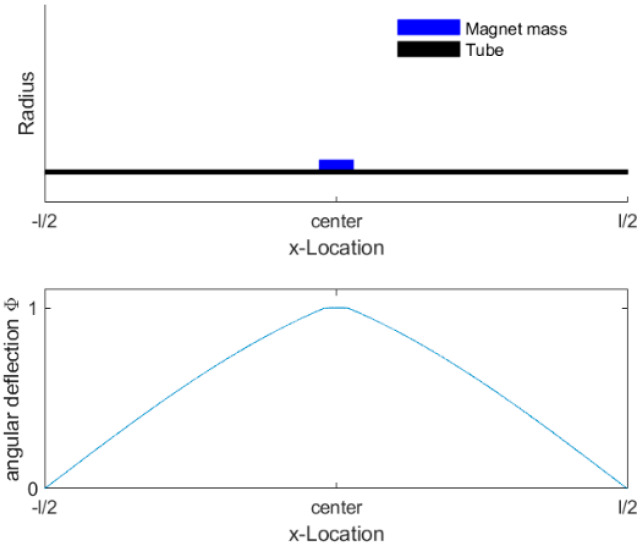
(**top**) Schematic cross-section of the tubular sensor, including the tube and magnet mass; (**bottom**) normalized solution of the excited torsional mode over measurement length ±l/2.

The sensor could be modeled as a classic harmonic resonator, where temporal and structural parts are independently considered. To compute the shape of the torsional mode, the equation for torsional waves in nonhomogeneous cylindrical structures is solved, with the contribution of the attached magnets considered in a simplified manner. The inertial mass of the magnets was modelled by a larger cylindrical section. This larger cylindrical section increases the internal mass to account for the additional inertial mass of the attached magnets and stiffens the section of the larger cylinder. This larger cylindrical section is shown in Figure 4 (top) by the “magnet mass”. The equation for torsional waves is shown as Equation (1),

(1) $$\frac{\partial }{\partial x}\left(GI_{p}\right)\cdot \frac{\partial \Psi }{\partial x}-2\pi R^{2}\tau +F=I_{p}\frac{\partial^{2}}{\partial t^{2}}\Psi$$

where Ψ*,* angular deflection; $I_{p}$, second moment area; x, axial direction; G, shear modulus; R, inner tube radius; F, excitation force; τ; viscous shear stress on the structure; and t, time.

We assumed that the solution of Equation (1) could be written by a space- and time-dependent function (see Equation (2)). Therefore, the temporal and structural parts could be solved independently.

(2) $$\Psi \left(x,t\right)=\varphi \left(t\right)\cdot \hat{\phi }\left(x\right)$$

To compute the shape of the structural mode, excitation and fluid forces were neglected. This weakly coupled fluid–structure interaction approach holds true for fluids with a low viscosity, where fluid-induced forces are much smaller than structural forces. At higher viscosities, the fluid may influence the shape of the structural mode. To compute the shape of the mode, we assumed that the angular deflection at the masses was zero because the moment of inertia was much higher than that of the tube. This defined the boundary conditions at the end of the measuring section (±l/2); see Equation (4).

(3) $$G\frac{\partial }{\partial x}\left(I_{p}\cdot \frac{\partial \hat{\phi }}{\partial x}\right)=-\omega^{2}I_{p}\rho \hat{\phi }$$

(4) $$\hat{\phi }\left(-\frac{l}{2}\right)=0,\;\;\;\;\hat{\phi }\left(+\frac{l}{2}\right)=0,\;\;\;\;\;\;\;\max\left(\hat{\phi }\right)=1$$

Equation (3) is a boundary value problem that could be solved numerically in MATLAB by using the bvp4c (fourth-order method for boundary value problems) function [32]. Thereby, only the solution of the first torsional mode was computed with its corresponding natural frequency, as shown in Figure 4. Due to the inertial load caused by the magnets (blue, Figure 4), the mode was distorted in the central section. This created large local bending of the modal function at the edge where magnets are attached to the tube and results in a slight straightening of the rest of the tube. The time-dependent component of the oscillation is represented by an ordinary differential equation. The representative viscous torque, the moment of inertia, and spring constant were obtained by integration over the length l, see Equations (6) and (7). 

The excitation term F was neglected because it was not present when the measurement took place. Thus, the resonator could be modeled by an ordinary differential (Equation (5)) under the assumption of a time-harmonic solution of $\left(t\right)=ℜ\left(\hat{X}e^{i\omega t}\right)$:

(5) $$\frac{\partial^{2}\varphi }{\partial t^{2}}J_{0}+\varphi \cdot c+{\hat{M}}_{v}\hat{X}e^{i\omega t}=0$$

(6) $$J_{0}=\overset{l/2}{\underset{-l/2}{\int }}\left[\rho \left|\hat{\phi }\left(x\right)\right|\cdot I_{p}\left(x\right)\right]dx$$

(7) $${\hat{M}}_{v}=\overset{l/2}{\underset{-l/2}{\int }}\frac{\hat{\tau }\left(x\right)2\pi R^{2}}{\hat{X}}dx$$

where $\hat{\phi }$, angular deflection; c, mode spring constant; $\hat{X}$, amplitude;$\;i=\sqrt{-1}$; ω, the angular frequency; and ${\hat{M}}_{v}$, fluid-induced torque. Using the time-harmonic assumption, we get Equation (8):

(8) $$-\omega^{2}J_{0}+c+{\hat{M}}_{v}=0$$

Equation (8) can be solved as an eigenvalue problem, where the eigenvalue λ=iω. On the basis of the solution, the bandwidth Γ of the resonator can be determined from the logarithmic decrement of the oscillation, which is the real part of the eigenvalue λ. Similarly, the angular resonance frequency $\omega_{0}\;$ is the imaginary part of λ.

(9) Γ=ℜ(λ)

#### Fluid Forces

The torsional oscillation of the tube created fluid motion, and thus shear stresses at the inner wall of the tube where the fluid is in contact with the solid. These shear stresses *τ* created a torque, which damped the oscillation. To compute the shear stresses, a simplified set of the linearized Navier–Stokes equation was solved. Flow within the tube was approximated under the assumption of no axial flow, no azimuthal change, and no radial flow. On the basis of these assumptions, a simplified version of the Navier–Stokes equation could be written in cylindrical coordinates, where u, azimuthal velocity; η, dynamic viscosity; ρ, fluid density; r, radius; and p, pressure—see Equations (10) and (11). This approach was already used by Fuchs and Drahm [31] for cylindrical geometries.

(10) $$\frac{\partial u}{\partial t}=\frac{\eta }{\rho }\left(\frac{1}{r}\frac{\partial u}{\partial r}+\frac{\partial^{2}u}{\partial r^{2}}+\frac{u}{r}\right)$$

(11) $$\frac{u^{2}}{r}=\frac{\partial p}{\partial r}$$

Then, we assumed a time-harmonic solution (Equation (12)).

(12) $$\hat{u}i\omega =\frac{\eta }{\rho }\left(\frac{1}{r}\frac{\partial \hat{u}}{\partial r}+\frac{\partial^{2}\hat{u}}{\partial r^{2}}+\frac{\hat{u}}{r}\right)$$

A solution to Equation (12) could be found (see Equation (14)), where $J_{1}$ was the Bessel function of the first kind, $Y_{1}$ the Bessel function of the second kind and $c_{1}$,$\;c_{2}$ coefficients.

(13) $$\hat{u}\left(r\right)=c_{1}\cdot J_{1}\left({\left(-1\right)}^{\frac{3}{4}}r\sqrt{\frac{\omega \rho }{\eta }}\right)+c_{2}\cdot Y_{1}\left(-{\left(-1\right)}^{3/4}r\sqrt{\frac{\omega \rho }{\eta }}\right)$$

Boundary conditions were $\hat{u}\left(r=0\right)=0$ and $\hat{u}\left(r=R\right)={\hat{v}}_{0}$, where R is the tube inner radius and ${\hat{v}}_{0}$ the wall velocity. The wall velocity depended on the axial location, as well as the rate of angular deflection; see Equation (14).

(14) $${\hat{v}}_{0}=\hat{X}r\omega \left|\hat{\phi }\left(x\right)\right|$$

The flow field can then be described by Equation (15).

(15) $$\hat{u}\left(r\right)={\hat{v}}_{0}\cdot \frac{J_{1}\left({\left(-1\right)}^{\frac{3}{4}}r\sqrt{\frac{\omega \rho }{\eta }}\right)}{J_{1}\left({\left(-1\right)}^{\frac{3}{4}}R\sqrt{\frac{\omega \rho }{\eta }}\right)}$$

**Figure 5:**
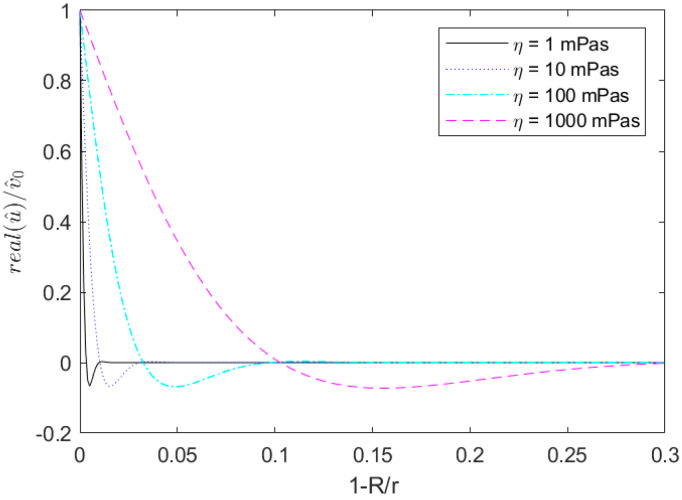
Velocity profile for different fluids in a tubular sensor with a frequency of 10,800 Hz and density of 1000 kg/m^3^.

Figure 5 shows the real part of the azimuthal velocity u for three different viscosities at a constant density of 1000 kg/m^3^. For all solutions, flow velocity was near zero within the first 30% of the radius; thus, any flow effects occur in the vicinity of the wall.

On the basis of Equation (15), shear rates and thus the viscous-induced damping could be determined. Viscous-induced torque ${\hat{M}}_{v}$ was computed by integrating shear stress $\hat{\tau }$ over the wall of the tube; see Equation (7). Shear stress was defined by Equation (16) at the radius of the inner wall R.

(16) $$\hat{\tau }\left(x\right)=\eta \left(\frac{\partial \hat{u}\left({\hat{v}}_{0}\left(x\right)\right)}{\partial r}-\frac{\hat{u}\left({\hat{v}}_{0}\left(x\right)\right)}{r}\right)$$

## 3. Discussion

The sensor was tested in two different stages. In the first stage, static experiments were conducted under well-defined conditions where the fluid properties were well known. These experiments were used to create a baseline for the sensor and validate the numerical model. The numerical model was then fitted to the experiments to account for any systematic deviation. The fitting was carried out by multiplying the prediction with an empirical correction function. This corrected prediction was then used to predict the fluid’s viscosity based on the measured properties. In the second stage, sensor sensitivity towards internal flow was evaluated by comparing the measured damping for the same fluids with and without internal flow.

### 3.1. Static Flow Conditions

**Figure 6:**
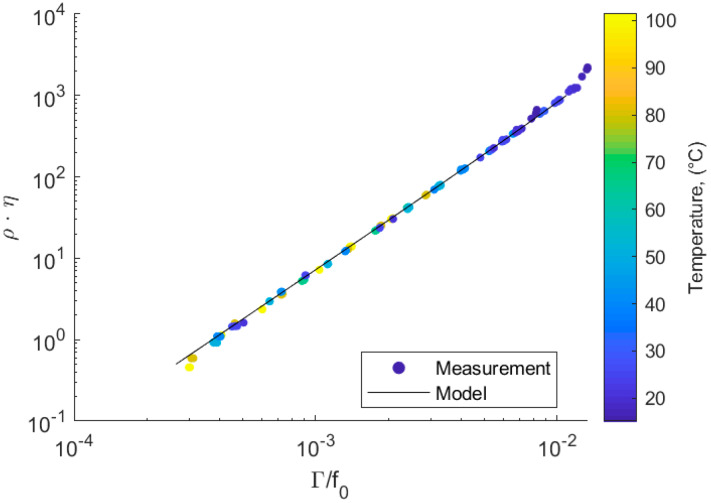
Numerical prediction and experimental measurements of the ρη value at different temperatures, where ρ is the density and η the viscosity.

Experiments were conducted under static, thermally uniform conditions using fluids with a well-known property. The measured fluid-induced damping versus the product of fluid denisty and viscosity (ρη) is shown in Figure 6. The colormap shows the temperature at which the measurement was conducted. To mitigate any temperature effects, the measured bandwidth was divided by the resonance frequency. This was carried out because the shear modulus of the resonator was temperature dependent. The resonance frequency and bandwidth of the sensor decreases with increasing temperature. By dividing the bandwidth by the resonance frequency, the temperature dependence of the damping could be compensated, and the measurements collapsed to a single line. Thus, the sensor measures the same $\Gamma /f_{0}$ value independent of fluid temperature, as can be seen in Figure 6. 

The model described in Section 2.4 enables the prediction of the ρη value, where ρ is the fluid density and η the dynamic viscosity. This prediction of the ρη value for a given damping is shown as a black line in Figure 6 (black line). The predictions were within the same order of magnitude and show the same trend as the experimental measurements. This indicates that the model captured the primary effects of the resonator. For small viscosities, the model predicted that damping increases proportionally to the square root of ρη, which is a typical property of sensors based on torsional resonators. This is the case, as long as the penetration depth $\delta =\sqrt{2\eta /\left(\rho \right)}$ is much smaller than the inner radius from the tube (2.625 mm). The penetration depth δ ranges from 0.054 mm at a dynamic viscosity of 1 mPas up to 0.171 mm for a viscosity of 1000 mPas. Thus, at higher viscosities, the curvature of the tube becomes relevant and the predicted damping relatively decreases. This effect is present in both simulation and experiment, but more predominant in the experiments. 

**Figure 7:**
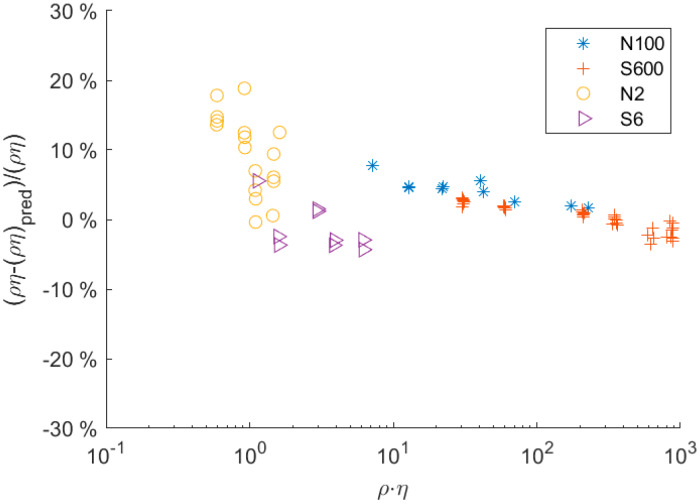
Relative deviation of the predicted and actual ρη  value, where ρ  is the density and η the dynamic viscosity.

The deviation between measurement and model is more evident in Figure 7. Despite the overall trend being in good agreement, predictions systematically differed for high ρη values. At low ρη values, there was a constant offset between numerical predictions and experiments, which could be explained by manufacturing tolerances. However, at high ρη values, i.e., high damping, there was systematic deviation in the trend. This systematic deviation was statistically significant and could be caused by an effect that was neglected in the model. Potential sources of the deviation include

(1) Bias in the damping measurement: At high damping, the signal-to-noise ratio (SNR) decreased due to the smaller amplitude of the resonator. The algorithm used to determine the damping was sensitive to the noise in the signal. As the SNR decreased, the error in the evaluation of the damping value increased. The error is not normally distributed but had a bias towards smaller damping values. Hence, the evaluated averaged value of the damping tended to be underpredicted as the SNR decreased. This behavior could be qualitatively simulated and showed a similar trend, as was experimentally observed.
(2) Distortion of modal function: Another potential source of the systematic deviation is the fluid–structure interactions. At high ρη values, the fluid exerts forces on the tube that are much higher than those exerted at low ρη values; thus, the balance between structural and fluid forces changes. In the model, the modal shape was computed under the assumption that the fluid forces did not impact the shape of the mode. Hence, this assumption may no longer be valid for fluids with high ρη values. To account for and verify this effect, the fluid–structure interaction (strong coupling) will be incorporated into the numerical model in future studies. This would allow specific investigation of the impact of fluid properties on the structural mode and its implications at ρη values.

To account for those effects which were not accounted for in the numerical prediction, an empirical polynomial model was used to correct the deviation between the predicted and measured values; see Equation (17). This polynomial was multiplied by the numerical prediction to correct the small deviations between the numerical prediction and experimental data. The multiplier function was a polynomial based on the log of the ρη value. Coefficients were determined by the least squares method on the relative deviation from prediction to measurement (Equation (18)).

(17) $$\frac{\Gamma }{f_{0}}\approx \frac{\Gamma_{num}\left(\rho \eta \right)}{f_{0,num}}\cdot \overset{4}{\underset{i=0}{\sum }}a_{i}\log{\left(\rho \eta \right)}^{i}$$

(18) $$a_{i}≔\min\left(\sum^{\text{​}}{\left[\frac{\frac{\Gamma_{num}\left(\rho \eta \right)}{f_{0,num}}\cdot \sum_{i=0}^{4}a_{i}\log{\left(\rho \eta \right)}^{i}-\frac{\Gamma }{f_{0}}}{\frac{\Gamma_{num}\left(\rho \eta \right)}{f_{0,num}}\cdot \sum_{i=0}^{4}a_{i}\log{\left(\rho \eta \right)}^{i}}\right]}^{2}\right)$$

**Figure 8:**
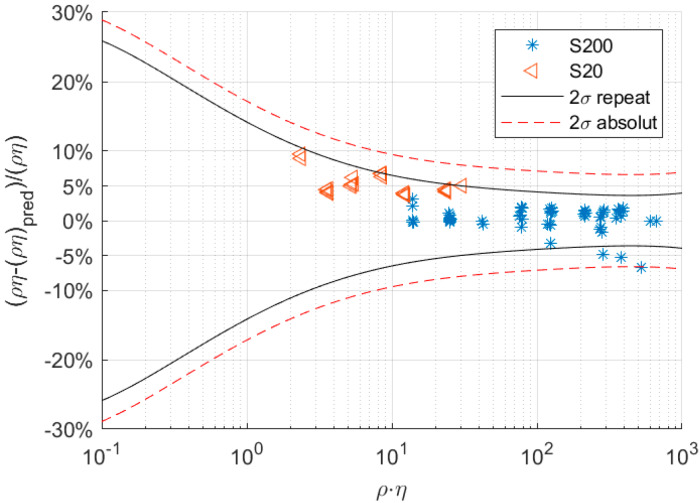
Relative deviation of the predicted and actual ρη value for the fluids S20 and S200, where ρ is the density and η is the dynamic viscosity. The full and dashed lines show the 95% confidence interval (2σ) for repeatability and absolute value, respectively.

To validate the baseline model, we tested it against two other viscosity reference fluids from Cannon, S20 and S200, which were not used to create a baseline for the sensor, i.e., to determine coefficient $a_{i}$. Therefore, the measured damping was used to determine the ρη value of the fluid (using Equation (17)). This predicted ρη value was then compared to the actual ρη value of the fluid used in the measurement. Figure 8 shows the relative deviation between the predicted (Equation (17)) and actual ρη value of the fluid. Deviation from the predicted to the actual ρη value was within the confidence interval. The black line indicates the 95% confidence interval in terms of repeatability, whereas the red line shows the respective 95% confidence level for predicting the absolute ρη value. The uncertainty of predicting the absolute ρη value was higher because it also contained the uncertainty of the basic calibration conducted in this study.

Overall, confidence intervals become smaller at higher ρη values and reach a minimum of ±4% for repeatability. 

### 3.2. Flow Loop

The sensor was tested in a flow loop to account for flow effects such as turbulent or laminar flow. This experiment was necessary to investigate the interaction between internal flow and flow induced by torsional vibration. This is important under turbulent conditions, where turbulences may interact and disturb the flow caused by the torsional vibration of the sensor and thus impact the measurement. This would create a flow or Reynolds dependence on the measurement. Experiments were conducted over a wide range of Reynolds numbers from 500 (laminar flow) up to fully turbulent conditions at 50,000. The variation in the Reynolds number was achieved by varying both flow rate and the fluid’s viscosity (by changing the glycerol concentration in water). 

**Figure 9:**
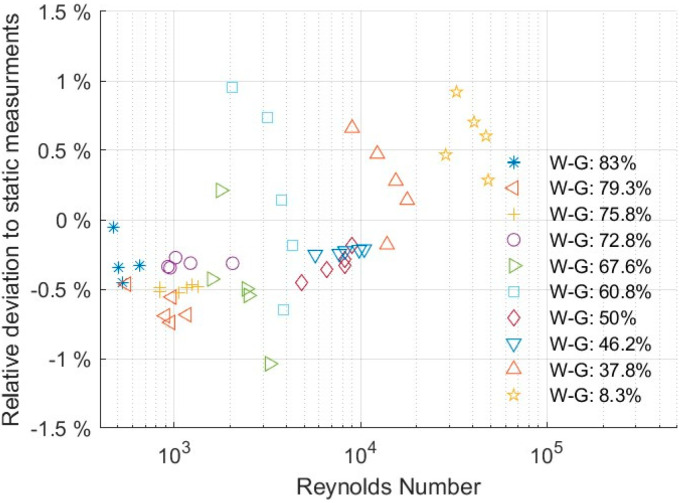
Relative deviation between static and flow measurements in a flow loop for different water–glycerol (W-G) concentrations.

Figure 9 shows the relative deviation of the predicted ρη value between static flow measurement and measurements with the internal flow. All deviations were below ±1%. This deviation was below the confidence interval of repeatability, and data were randomly spread. Hence, flow conditions shown in Figure 9 exhibited no significant influence on measurements of Reynolds numbers up to 50,000. Any variations were within the uncertainty of repeatability. 

## 4. Conclusions

We presented an experimentally validated numerical model for a nonintrusive, real-time, tubular sensor and tested for different viscosities and densities. The sensor was comprised of a straight tube and could be directly integrated into a piping system. The numerical model describing the sensor was derived on the basis of the torsional vibration of the tube and the interaction with the fluid inside the tube. The fluid interaction with the resonator was computed using an analytical fluid model. The modelled predictions were compared with four different fluids at temperatures between 20 and 100 °C and were found to be in good agreement at low viscosities. However, at high viscosities, there was systematic deviation between numerical prediction and experimental data. This deviation was likely caused by fluid-induced modal distortion or bias in the measurement error.

In order to account for the systematic deviation between prediction and measurement, the numerical prediction was multiplied with an empirical model. After this correction, the model was tested against two additional fluids. Measurements were in good agreement with the prediction and within the confidence interval. 

Additionally, the tubular sensor was tested in a flow loop with different water–glycerol solutions, simulating industrial conditions, in a Reynolds number range of 500–50,000. The sensor did not exhibit any Reynolds dependence. Overall, the tubular sensor showed good potential for application in industrial processes. However, further studies are needed to elucidate the departure of the model prediction from real sensor behavior at high viscosities.
